# A Comparative Analysis of Depressive Symptoms Following Sports-Related Concussion in Youth Athletes Versus Their Age-Matched Non-concussed Counterparts

**DOI:** 10.7759/cureus.28549

**Published:** 2022-08-29

**Authors:** Emily M Robinson, Sananthan Sivakanthan, Sharon Durfy, Frederick P Rivara, Sara Chrisman, Christine L Mac Donald

**Affiliations:** 1 School of Medicine, University of Washington, Seattle, USA; 2 Department of Neurological Surgery, University of Washington, Seattle, USA; 3 Department of Pediatrics, Seattle Children's Hospital, Seattle, USA

**Keywords:** brain injuries, phq-9, adolescent, pediatric, persistent post-concussive symptoms, depressive symptoms, mild traumatic brain injury, sports-related concussion

## Abstract

Background and objective

Athletics is the leading cause of pediatric concussion, and depression is a major comorbidity associated with concussion in the pediatric population. Prior studies have described the risk of depression after concussion in high school-, collegiate-, and elite-level athletes, but there is scarce data on younger athletes. Interpretation of existing research on the association of depression with concussions in youth athletes is complicated by diverse study designs, varying measures of depression, differing timelines for symptom development, and a lack of control groups. Furthermore, limited research exists on sex-related differences in the development of depressive symptoms following sports-related concussions (SRC) in younger athletes. This study used the Seattle Pediatric Concussion Research Collaborative (SPCRC) Data Repository to compare depressive symptoms between youth athletes at one month post-SRC and non-concussed age-matched controls by using a standardized measure of depressive symptoms: the Patient Health Questionnaire-9 (PHQ-9). The secondary goal was to compare PHQ-9 scores between males and females for both concussed and non-concussed groups.

Methods

This study entailed a secondary analysis of data collected as part of the SPCRC Data Repository. We conducted a retrospective subgroup analysis of PHQ-9 scores at one month post-concussion for concussed youth athletes. We compared the PHQ9 scores of concussed youth athletes with PHQ-9 scores collected at the time of enrollment for non-concussed youth athletes.

Results

After random age-matching, a cohort of 266 patients (133 in the concussed group and 133 in the non-concussed control group) was included in the final analysis. The mean age was 13.8 years (range: 5-18 years). For the concussed group, a history of SRC was associated with a higher mean total PHQ-9 score at one month post-concussion compared with the control group at the time of enrollment (6.14 ±5.46 versus 1.53 ±1.81, respectively, p<0.0001). All nine subdomains of the PHQ-9 showed significantly higher scores in the concussion group compared with the control group (p<0.0001). Significantly higher scores were observed when comparing mean total PHQ-9 scores for male athletes in the concussion group with male athletes in the control group (7.03 ±5.72 versus 1.59 ±1.66, p<0.0001) and for female athletes in the concussion group compared with female controls (5.28 ±5.10 versus 1.49 ±1.92, p<0.0001). No significant differences were observed between sexes for total PHQ-9 scores or PHQ-9 subscores.

Conclusion

At one month post concussion, youth with SRC demonstrated higher levels of depressive symptoms as measured by PHQ-9 compared with age-matched typically developing controls. No significant differences were identified in total PHQ-9 scores and subscores between male and female participants for either the concussion or control group. This study suggests that clinicians need to be vigilant and monitor for symptoms of depression in young athletes for at least one month post-concussion.

## Introduction

Approximately 1.1 to 1.9 million sports-related concussions (SRC) occur annually in the United States among youth aged 18 years or younger [1]. A wide range of symptoms is commonly reported following SRC, including headache, cognitive dysfunction, and mood changes [2]. While often debilitating, these symptoms tend to be self-limiting, and the majority of patients achieve complete resolution of their symptoms within two to three weeks [3]. However, prior studies have shown that compared with adults, children and adolescents may be particularly susceptible to prolonged symptoms after concussion [4,5]; an estimated 14% of children will exhibit persistent symptoms three months later [6]. Such persistent symptoms after concussion in young athletes are collectively termed persistent post-concussive symptoms (PPCS) [7]. PPCS can be associated with psychological symptoms, including depression and anxiety [3,8], which may contribute to longer recovery time [9].

Prior studies have described the risk of depression after concussion in high school- [2,10], collegiate- [11,12], and elite-level athletes [13]. Two retrospective studies involving adolescents found that a history of concussion was associated with an increased risk of depression [8,14]. One prospective study by O’Connor et al. found a slight increase in the number of adolescent patients diagnosed with major depressive disorder in mild traumatic brain injury (mTBI) compared with patients with arm injuries at two years post-injury (5% versus 0%) [15]. Interpretation of existing research is complicated by variations in study designs, differing timelines regarding the development of depressive symptoms, diverse methods for measuring depression, as well as a lack of control groups. Furthermore, while there are studies examining sex-related differences in symptom burden and trajectory of recovery following concussion in other populations [16,17], there is limited data on sex-related differences in the development of depressive symptoms following SRC in younger athletes.

The primary aim of this research was to employ the Seattle Pediatric Concussion Research Collaborative (SPCRC) Data Repository to compare depressive symptoms between youth athletes at one month post-SRC and non-concussed age-matched controls by using a standardized measure of depressive symptoms: the Patient Health Questionnaire-9 (PHQ-9). The secondary goal was to assess for any confounding effect of sex on the severity of depressive symptoms.

## Materials and methods

Study cohort

This was a retrospective study conducted as a secondary analysis of data from the SPCRC Data Repository. The SPCRC Data Repository consists of aggregated and de-identified demographic and clinical outcomes data from six prospective, observational studies, some longitudinal in nature, of which four have published research findings to date [18-21]. For this research, we included data obtained from participants across all six studies. Study participants included youth across a broad age range (5-18 years) as reflected in the initial study enrollment. The SPCRC Data Repository was utilized to perform a subgroup analysis on symptoms of depression by utilizing PHQ-9 data collected at one month post-injury for five of six studies in the repository.

Recruitment procedures are described in detail elsewhere [19]; however, in general, study participants were screened for recruitment through common pathways including the Seattle Children’s Hospital (SCH) Emergency Department, SCH Sports Medicine Clinic, SCH Rehab Medicine Clinic, and also by screening community sports leagues (soccer and football). For the original six SPCRC studies, controls were identified from the same sports teams as concussed athletes, and study flyers and advertisements were posted through research networks at SCH and the University of Washington Institute of Translational Health Sciences. Consent was obtained from parents and assent from youth participants. Consent forms stipulated that de-identified data could be used for future secondary analyses.

For this report, a review was provided by the University of Washington Human Subjects Division (STUDY00003410), which determined that this research did not involve human subjects as defined by federal and state regulations. This determination was made because the use of de-identified data from the SPCRC Data Repository comprised secondary data aggregation and analysis of data from previous Institutional Review Board-approved studies, all of which included language about future de-identified data sharing in the original consent process.

The SPCRC Data Repository was queried to establish two patient groups: (1) concussed patients who had sustained a concussion during sports or recreational play (concussion group) and (2) typically developing youth with no history of prior head injury diagnosis, including concussion (control group). Previous diagnosis of concussion was not an exclusion criterion for the concussion group, whereas controls were excluded if they had any prior head injury diagnosis including concussion. For the concussed group, youth athletes with a PHQ-9 completed at one month post-injury were included if they had a diagnosis of SRC, onset or increase in at least three post-concussive symptoms following head impact, and post-concussive symptoms that persisted for at least four weeks after injury. Concussed patients were excluded if they had a history of prior neurological disease, psychiatric diagnoses, or traumatic brain injury (TBI) defined as any head injury exposure greater than a concussion. The control group included youth athletes with a PHQ-9 completed at the time of enrollment, with no concussion, no history of TBI including concussion, and no history of prior neurological disease or psychiatric diagnoses.

Patient Health Questionnaire-9

The PHQ-9 is a validated [22] instrument for screening, diagnosis, and monitoring of the severity of depression (see Appendices). This scale has demonstrated reliability and validity among both adults and youth [23,24]. The PHQ-9 incorporates the Diagnostic and Statistical Manual of Mental Disorders, Fourth Edition (DSM-IV) diagnostic criteria and rates the frequency of symptoms as a self-reported severity index. Index subscores are tallied for a total score indicative of depression severity. The nine subscores are as follows: 1. Little interest or pleasure in doing things; 2. Feeling down, depressed, or hopeless; 3. Trouble falling or staying asleep, or sleeping too much; 4. Feeling tired or having little energy; 5. Poor appetite or overeating; 6. Feeling bad about yourself or that you are a failure or have let yourself or your family down; 7. Trouble concentrating on things, such as reading the newspaper or watching television; 8. Moving or speaking so slowly that other people could notice or being so fidgety or restless that you have been moving around a lot more than usual; and 9. Having thoughts that you would be better off dead, or those of hurting yourself. The scores range from 0-27 with the following cutoff points, which have been previously described [22,24]: no depressive symptoms (0-4), mild depressive symptoms (5-9), moderate depressive symptoms (10-14), moderately severe depressive symptoms (15-19), and severe depressive symptoms (≥20).

Statistical analysis

**Figure 1:**
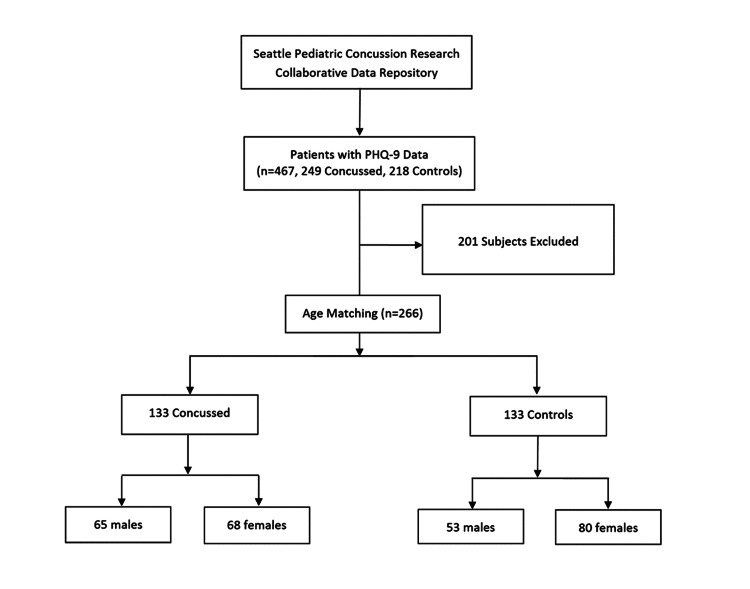
Consort diagram illustrating study enrollment and allocation to concussed and non-concussed groups PHQ-9: Patient Health Questionnaire-9

Study participants were selected from the repository based on age, sex, and PHQ-9 individual subdomains and total scores at the one-month follow-up. The ages of the groups were analyzed using a two-sided Mann-Whitney U test; a statistically significant difference was identified (mean of 14.45 years for the concussion group versus 13.17 years for the control group, p<0.0001). The two groups were subsequently age-matched randomly in a one-to-one fashion, with one control participant matched for one case based on age, in order to remove age as a confounder. Ages were rounded off to the nearest year for matching. In the event of a duplicate case or duplicate control, one of the subjects was removed from the analysis in a random fashion. The resultant age-matched groups were used in all subsequent analyses (Figure 1).

Data distributions by the group for each dataset of comparison were initially analyzed using a Shapiro-Wilk test for normality, which identified non-parametric distributions in some of the groups. Given this determination, all analyses were carried out using two-sided Mann-Whitney U tests followed by Bonferroni correction for multiple comparisons with significance assessed at p<0.05.

## Results

Participant selection and group composition

Data relating to 467 participants were initially extracted from the repository, including 249 patients in the concussion group and 218 participants in the control group (Figure 1). The groups were subsequently age-matched randomly, yielding a total cohort of 266 subjects with 133 in each group. The mean age was 13.8 years for each group. The concussion group consisted of 65 male and 68 female participants, and the control group included 53 male and 80 female participants.

Concussion and control group mean PHQ-9 totals and subscores

The mean total PHQ-9 scores and subscores for each of the nine PHQ-9 domains for both groups are shown in Table 1.

**Table 1:** Concussed group and non-concussed (control) group PHQ-9 scores and subscores *Denotes statistical significance after Bonferroni correction for multiple comparisons PHQ-9: Patient Health Questionnaire-9; SD: standard deviation

PHQ-9	Concussed, mean ±SD (n=133)	Non-concussed, mean ±SD (n=133)	P-value
Total	6.14 ±5.46	1.53 ±1.81	<0.0001*
Subscore 1	0.57 ±0.83	0.17 ±0.45	<0.0001*
Subscore 2	0.57 ±0.84	0.11 ±0.36	<0.0001*
Subscore 3	1.12 ±1.03	0.36 ±0.61	<0.0001*
Subscore 4	1.18 ±1.01	0.46 ±0.58	<0.0001*
Subscore 5	0.60 ±0.89	0.14 ±0.41	<0.0001*
Subscore 6	0.55 ±0.89	0.13 ±0.43	<0.0001*
Subscore 7	0.88 ±0.96	0.11 ±0.40	<0.0001*
Subscore 8	0.47 ±0.86	0.04 ±0.23	<0.0001*
Subscore 9	0.20 ±0.54	0.01 ±0.09	<0.0001*

The concussion group showed a significantly higher mean PHQ-9 at one month post-concussion compared with the control group's PHQ-9 scores at the time of enrollment (6.14 ±5.46 versus 1.53 ±1.81, respectively, p<0.0001). All nine subdomains of the PHQ-9 showed significantly higher scores for the concussion group compared with the control group (p<0.0001).

For both groups, PHQ-9 subscores were used to calculate severity ranges for depressive symptoms, from no depressive symptoms (0-4) to severe depressive symptoms (≥20) (Table 2).

**Table 2:** PHQ-9 severity range scores for depressive symptoms in concussed and non-concussed groups *Analyses carried out using two-sided Mann-Whitney U tests for none and mild depression symptom severity groups. **Analyses carried out using one-sample t-test for moderate through severe depression symptom severity groups PHQ-9: Patient Health Questionnaire-9

PHQ-9 depressive symptoms severity range	Concussed, n (%) (n=133)	Non-concussed, n (%) (n=133)	P-value
None (0-4)*	68 (51)	125 (94)	0.0003
Mild (5-9)*	33 (25)	8 (6)	0.61
Moderate (10-14)**	23 (17)	0 (0)	<0.0001
Moderately severe (15-19)**	5 (4)	0 (0)	<0.0001
Severe (≥20)**	4 (3)	0 (0)	<0.0001

A total of 68 (51%) patients in the concussion group had PHQ-9 scores falling in the range for no depressive symptoms, compared with 125 (94%) patients in the control group; the difference was statistically significant (p=0.0003). A total of 33 (25%) patients in the concussion group met the criteria for mild depressive symptoms, compared with eight (6%) subjects in the control group. The category of mild depressive symptoms was the highest depressive symptom severity range calculated for the control group. In contrast, a total of 65 (49%) patients in the concussion group had depressive symptom severity scores ranging from mild to severe, including 33 (25%) mild, 23 (17%) moderate, five (4%) moderately severe, and four (3%) severe.

Concussion and control group mean PHQ-9 totals and subscores by sex

Analysis of PHQ-9 scores and subscores by sex for concussion and control groups are shown in Table 3.

**Table 3:** PHQ-9 total and subscores for male and female concussed and non-concussed subjects *Denotes statistical significance after Bonferroni correction for multiple comparisons PHQ-9: Patient Health Questionnaire-9; SD: standard deviation

PHQ-9	Male concussed, mean ±SD (n=65)	Male non-concussed, mean ±SD (n=53)	P-value	Female concussed, mean ±SD (n=68)	Female non-concussed, mean ±SD (n=80)	P-value
Total	7.03 ±5.72	1.59 ±1.66	<0.0001*	5.28 ±5.10	1.49 ±1.92	<0.0001*
Subscore 1	0.66 ±0.91	0.19 ±0.40	0.002*	0.49 ±74	0.15 ±48	0.0001*
Subscore 2	0.71 ±0.88	0.08 ±0.27	<0.0001*	0.44 ±78	0.14 ±41	0.003*
Subscore 3	1.22 ±1.08	0.36 ±0.65	<0.0001*	1.03 ±98	0.36 ±58	<0.0001*
Subscore 4	1.35 ±1.04	0.55 ±0.54	<0.0001*	1.02 ±95	0.40 ±61	<0.0001*
Subscore 5	0.71 ±0.96	0.15 ±0.36	0.0003*	0.50 ±80	0.14 ±44	0.0003*
Subscore 6	0.68 ±0.95	0.11 ±0.32	<0.0001*	0.43 ±82	0.14 ±50	0.004*
Subscore 7	0.98 ±0.99	0.11 ±0.38	<0.0001*	0.78 ±93	0.11 ±42	<0.0001*
Subscore 8	0.48 ±0.87	0.04 ±0.19	0.0002*	0.46 ±85	0.04 ±25	<0.0001*
Subscore 9	0.25 ±56	0.00	0.001*	0.15 ±53	0.01 ±11	0.03

The mean total PHQ-9 score for males in the concussion group was 7.03 ±5.72 compared to 1.59 ±1.66 for males in the control group; the mean total PHQ-9 score for females in the concussion group was 5.28 ±5.10 compared to 1.49 ±1.92 for females in the control group. These differences in total PHQ-9 scores were statistically significant for both males and females (p<0.0001).

As shown in Table 3, all PHQ-9 subscores were significantly different when comparing males in the concussion group with males in the control group (p≤0.002). For female study participants, all PHQ-9 subscores were also significantly different between the concussion and control groups, except for subscore 9 (p=0.03, not significant after Bonferroni correction). After correction for multiple comparisons, there were no statistically significant differences observed for either concussed or control groups between sexes for PHQ-9 total score or subscores (p-value ranging from 0.05 to 0.13 for the concussed group; p-value ranging from 0.07 to 0.99 for the control group).

## Discussion

Based on data from the SPCRC Data Repository, our study shows that youth with SRC had a statistically significant higher mean total PHQ-9 score at one month post-concussion compared with PHQ-9 scores of their age-matched, non-concussed counterparts. These results are similar to previous research indicating an association between concussion and post-injury depressive symptoms in children and adolescents [8,14,25,26]. Furthermore, nearly half (49%) of the youth in our study concussion group met the PHQ-9 criteria for depressive symptoms ranging from mild to severe, compared with 6% of youth controls, underscoring the relationship between concussion and depressive symptoms.

One prior study that undertook a retrospective analysis of the National Survey of Children's Health has reported on the prevalence of depression in concussed adolescents [14]. Other retrospective studies examining the association between depression after concussion with various risk factors have compared athletes’ baseline depression symptoms with post-concussion symptom testing [2,8,10,11]. While some of these studies include large numbers of study participants, these study designs do not include non-concussed controls. In a study of 84 NCAA Division 1 collegiate athletes with diagnosed concussions, Vargas et al. employed Beck Depression Inventory scores for both pre- and post-concussion and included a non-concussed control group [12]. However, in contrast to our research using age-matched controls, the Vargas et al. study used 44 undergraduate students who were not age-matched to concussed athletes for their control group.

Identifying, understanding, and treating post-concussion mental health symptoms, including depressive symptoms, is critical in youth athletes because children and adolescents with psychiatric symptoms and/or disorders are at increased risk of additional long-term comorbidities such as poor academic performance [25], substance abuse [27], and suicidal behavior [28]. Additionally, the persistence of post-concussion depressive symptoms is of considerable clinical importance. A longitudinal study in youth aged 10-14 years found that 50% of concussed patients met the criteria for depressive symptoms on PHQ-9 at six months post-injury, despite considerable improvements in cognitive domains [19]. These results contrast with recent findings from a longitudinal prospective study of depressive symptoms in high-school athletes with SRC. Hammer et al. found that symptoms of depression scored using the PHQ-9 worsened at 24-72 hours and also at seven days post-concussion, but improved by the time of return to sport (median duration of 14 days) and remained steady until at least 12 months after injury [2]. While these data contrast with our results of elevated PHQ-9 at one month post-injury, the mean age of the Hammer et al. study population was 16.3 years compared with 13.8 years in our study. Previous research has shown that younger populations are more susceptible to long-term symptoms from concussion [4,5], perhaps contributing to the elevated PHQ-9 scores at one month post-concussion in our younger study population. In addition, Hammer et al. recruited athletes who had sustained a concussion that was reported to athletic trainers, possibly leading to a cohort with less severe symptoms that also may have been less likely to be persistent. In our study, all athletes were recruited from outpatient subspecialty clinics and hence may have been more likely to have more severe, persistent depressive symptoms or additional comorbidities.

Previous studies of sex-related differences in depressive symptoms after SRC in adolescent athletes have demonstrated inconsistent findings. In this research, we found that mean total PHQ-9 scores for male and female youth athletes at one month post-concussion were statistically higher compared with their age-matched controls. However, we observed no significant differences in total PHQ-9 scores and subscores between male and female participants for either the concussion or control group. Ellis et al. reported that patients (mean age: 14.2 years) who developed post-concussive mental health problems were significantly more likely to be female [8]. They used the Post-Concussion Symptom Scale and clinical interview findings at the initial consultation (median of nine days post-concussion), and at one- to four-week intervals, for this research. Another study investigating depression-specific symptoms after concussion in a cohort of high-school and collegiate athletes found no evidence of sex-related differences in depression levels, using the Beck Depression Inventory-II up to 14 days post-concussion [16]. One meta-analysis concluded that female athletes across diverse age groups (middle school to collegiate athletes) tended to endorse greater overall depression symptoms post-concussion, and tended to experience longer times to symptom resolution compared with male athletes; however, none of these trends were statistically significant [29]. Thus, the impact of athlete sex on depression symptoms following SRC remains unclear in youth athletes, warranting additional research.

This retrospective study has a few limitations, including the potential for selection, reporting, and recall bias. Another limitation is the lack of baseline PHQ-9 scores for all concussed youth athletes. It is possible that some athletes had depressive symptoms or undiagnosed mental health disorders prior to their injury. Research conducted by the initial studies attempted to address this through careful screening for prior history of mental illness as part of patient evaluations, and patients were excluded if there was a history of prior neurological disease, brain injury, or a diagnosed mental health disorder. Due to the retrospective design of this review, we were unable to control for potentially confounding variables such as type of sport (football, soccer), level of the sport (community league or premier level), or situational risk factors that might have independently contributed to increased depression or undiagnosed mental health disorders. Furthermore, due to the considerable variation in the demographics collected across the initial studies, a formal multivariate analysis was not performed. This study provides data on PHQ-9 scores obtained one month after concussion and did not assess longer time periods post-concussion. The use of the PHQ-9 may have also caused certain limitations because it is a self-reported questionnaire with subscores that overlap with typical post-concussive symptoms. However, the PHQ-9 has demonstrated reliability and validity in both adults and youth [23,24] and is a commonly used tool in healthcare settings.

## Conclusions

Sports-related concussion in youth athletes is an important public health issue. In this study, at one month post-concussion, youth with SRC demonstrated higher levels of depressive symptoms as measured by PHQ-9 compared with age-matched typically developing controls. No significant differences were identified in total PHQ-9 scores and subscores between male and female participants for either the concussion or control group. This study shows that depressive symptoms in youth athletes who have suffered a concussion may persist, suggesting that clinicians need to be vigilant and monitor for symptoms of depression in youth athletes for at least one month post-concussion.

## Figures and Tables

**Figure 2:**
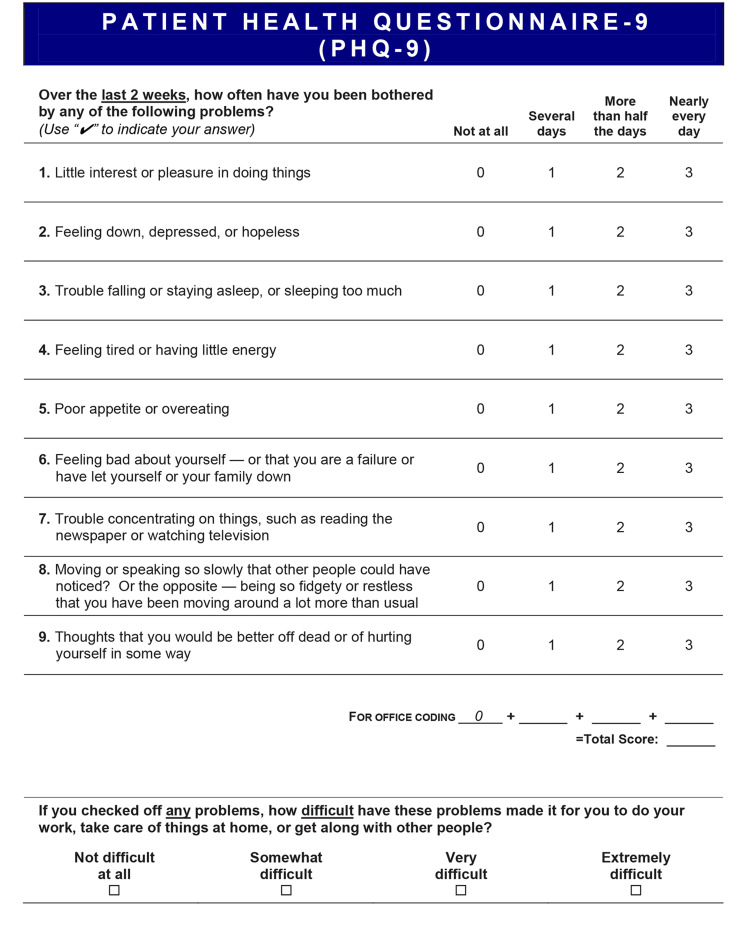
Patient Health Questionnaire-9 (PHQ-9) Developed by Drs. Robert L. Spitzer, Janet B.W. Williams, Kurt Kroenke and colleagues, with an educational grant from Pfizer Inc. No permission required to reproduce, translate, display, or distribute this image
